# Case Report: Proteinase 3 Antineutrophil Cytoplasmic Antibody-Associated Ulcerative Colitis Presenting as Recurrent Intestinal Pseudo-Obstruction in a Teenage Patient With *in situ* Proteinase 3 Immunohistochemical Staining

**DOI:** 10.3389/fped.2022.822491

**Published:** 2022-02-23

**Authors:** Ching-Wen Yang, Yu-Chien Kao, Pei-Chun Lin, Hsi-Yuan Chien, Sheng-Chieh Lin, Yu-Hsien Lee, Yen-Lin Huang, Shiuh-Bin Fang

**Affiliations:** ^1^Division of Gastroenterology, Hepatology, and Nutrition, Department of Pediatrics, Shuang Ho Hospital, Taipei Medical University, New Taipei City, Taiwan; ^2^Department of Pediatrics, School of Medicine, College of Medicine, Taipei Medical University, Taipei, Taiwan; ^3^Department of Pathology, Shuang Ho Hospital, Taipei Medical University, New Taipei City, Taiwan; ^4^Department of Gastroenterology, Shuang Ho Hospital, Taipei Medical University, New Taipei City, Taiwan; ^5^Department of Gastroenterology, Taipei Medical University Hospital, Taipei, Taiwan; ^6^Division of Allergy, Immunology, and Rheumatology, Department of Pediatrics, Shuang Ho Hospital, Taipei Medical University, New Taipei City, Taiwan; ^7^Department of Medical Imaging, Shuang Ho Hospital, Taipei Medical University, New Taipei City, Taiwan; ^8^Master Program in Clinical Genomics and Proteomics, College of Pharmacy, Taipei Medical University, Taipei, Taiwan

**Keywords:** antinuclear antibody (ANA), intestinal pseudo-obstruction, proteinase 3 antineutrophil cytoplasm antibody (PR3-ANCA), ulcerative colitis (UC), *in situ* immunohistochemical staining

## Abstract

Ulcerative colitis (UC) is a chronic relapsing inflammatory bowel disease with the colorectum as its major target organ. Involvement of the upper gastrointestinal tract in UC is rare and presents with nonspecific endoscopic and microscopic characteristics. Recent studies have demonstrated proteinase 3 antineutrophil cytoplasmic antibody (PR3-ANCA) to be a serological marker for differentiating UC from Crohn's disease in children and for detecting disease activity and nonresponse to steroid therapy and antitumor necrotizing factor-α agents. Herein, we report a 13-year-old female patient mainly presenting with recurrent bilious vomiting who was initially diagnosed with acute gastroenteritis. Intestinal pseudo-obstruction was confirmed through observation of a patent but segmentally dilated jejunum in the barium follow-through examination and other imaging; such obstruction can be attributed to backwash ileitis, superior mesenteric artery syndrome, ileus due to hypokalemia, or PR3-associated enteritis. Laboratory data revealed leukocytosis with neutrophil predominance and serum antinuclear antibody and PR3-ANCA positivity. Overlapping syndrome with autoimmune diseases was suspected. Pathology revealed a crypt abscess with aggregates of neutrophils consistent with UC but did not indicate vasculitis. The *in situ* immunohistochemical staining revealed PR3 density mainly in the colon and focally in the duodenum. To our knowledge, this is the first case report with *in situ* pathological evidence of PR3 in inflamed intestinal tissues in a patient with UC and with rare initial presentation of intestinal pseudo-obstruction–induced recurrent bilious vomiting. Whether the clinical features of the present case constitute overlap syndrome with other autoimmune disease or a disease variation of UC warrants further investigation. Notably, the patient's serum PR3-ANCA titers remained high in coincidence with increased disease activity and nonresponse to steroid therapy, but became lower after infliximab treatment. PR3-ANCA as a potential serum biomarker to aid in making differential diagnoses of UC in children, correlating disease activity, and predicting therapeutic responses was also reviewed.

## Introduction

Ulcerative colitis (UC) is a global disease with an incidence that has accelerated in Eastern Asia during the twenty-first century (1). UC is a chronic relapsing inflammatory bowel disease (IBD) with the colorectum as its major target organ (2); it presents as bloody diarrhea, rectal bleeding, abdominal pain, and extraintestinal manifestations. However, the involvement of the upper gastrointestinal (UGI) tract in UC is rare and has nonspecific endoscopic and microscopic characteristics (2); for diagnosis of UC, clinical, laboratory, radiological, endoscopic, and histopathological criteria must be met. Antineutrophil cytoplasmic antibodies (ANCAs), including proteinase 3 (PR3)-ANCA (or cytoplasmic ANCA [c-ANCA]) and myeloperoxidase (MPO)-ANCA (or perinuclear ANCA [p-ANCA]) (3), have been investigated as potential serum biomarkers to aid in making diagnoses, correlating disease activity, and predicting therapeutic responses. In Western countries, MPO-ANCA has been reported to be useful in differentiating UC from Crohn's disease (CD) due to its 65% positivity rate in patients with UC compared with a <10% positivity rate in patients with CD (4). However, in Japanese patients with UC, PR3-ANCA was found to be more commonly positive (39.2%−53.5%) than MPO-ANCA (3, 5). The positivity rate of PR3-ANCA in pediatric patients with UC was higher (57.6%) than that in adult patients with UC (6). Therefore, PR3-ANCA may be a more valuable serological marker for differential diagnosis of UC in children than MPO-ANCA is (7). Nevertheless, *in situ* pathological evidence of PR3 in patients with UC has not been reported.

## Case Report

**Figure 1:**
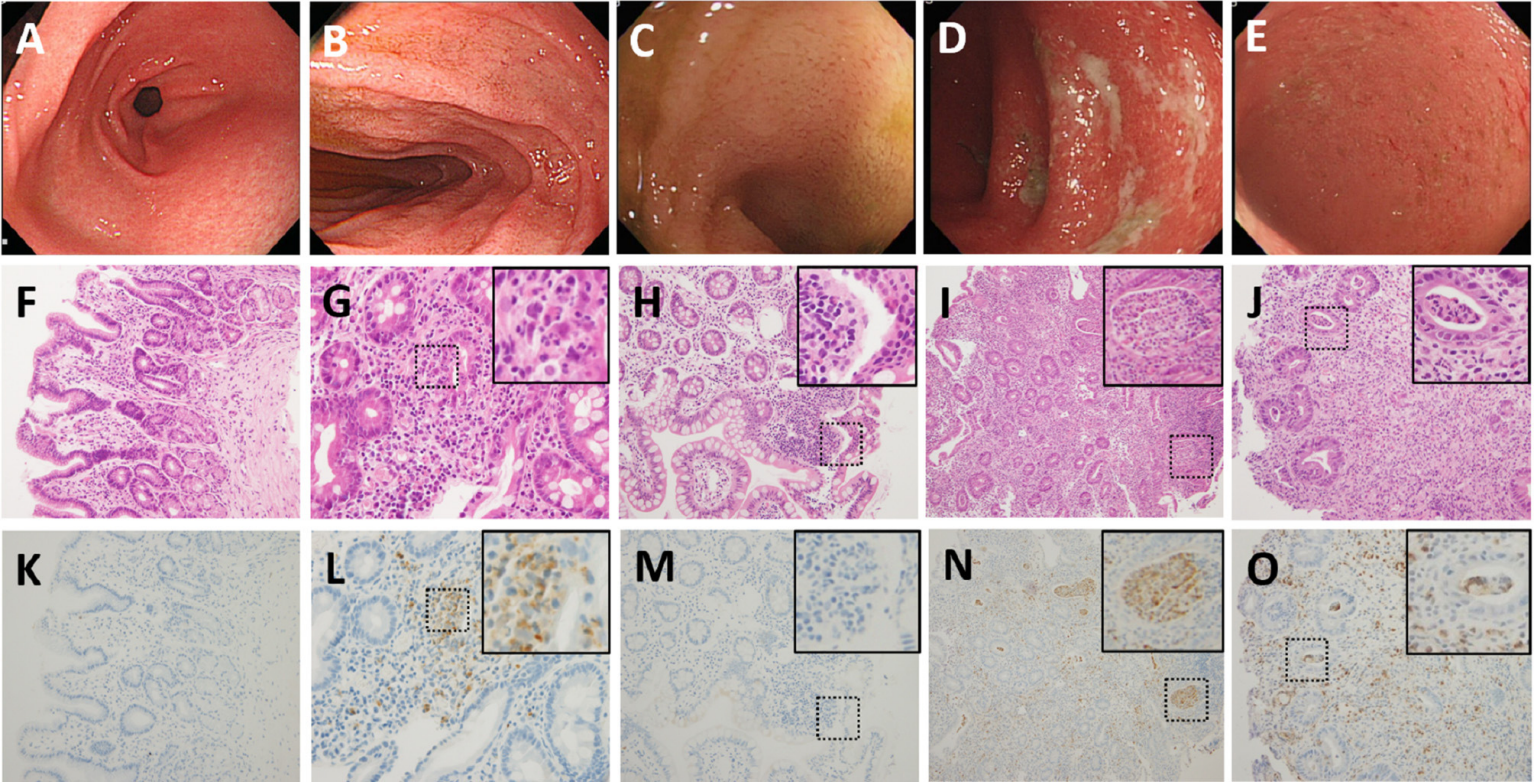
Representative biopsy site-oriented images of esophagogastroduodenoscopy, histopathology, and *in situ* PR3 immunohistochemical staining during the third and fifth hospitalizations. Esophagogastroduodenoscopy revealed hyperemic mucosa with hyperemic spots in **(A)** gastric antrum and **(B)** dilated duodenum lumen compatible with superficial gastritis and distal obstruction; colonoscopy revealed hyperemic edematous mucosa in the **(C)** terminal ileum, diffuse ulcerations of mucosa with exudates and easy touch bleeding in the **(D)** ascending colon and **(E)** sigmoid colon. Histopathology through hematoxylin and eosin staining and PR3 immunohistochemical staining (magnification power 200×) of biopsies revealed chronic inflammatory infiltrates and distorted glandular architecture in the **(F,K)** gastric antrum, **(G,L)** duodenum, **(H,M)** terminal ileum, **(I,N)** ascending colon, and **(J,O)** sigmoid colon with locally magnified insets (400×) revealing PR3-positive neutrophils focally stained in the **(L)** duodenal mucosa and densely stained in the mucosa of ascending and sigmoid colons, especially in **(N,O)** crypt abscesses, but sparsely in the **(M)** terminal ileum.

**Figure 2:**
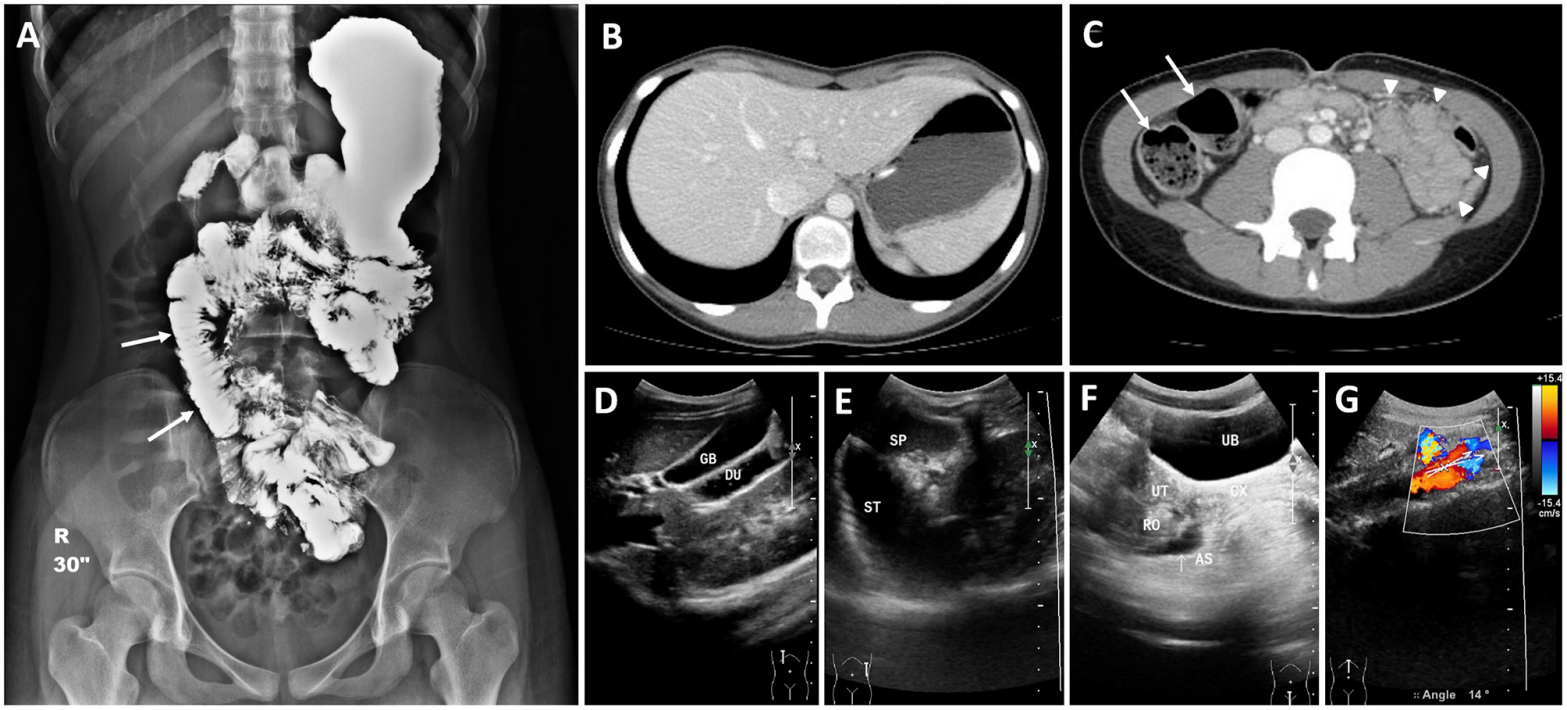
Medical images during third hospitalization of patient with recurrent bilious vomiting. **(A)** Barium follow-through examination revealed patency in the gastrointestinal tract but segmental dilation at the distal jejunum (arrows), which is compatible with intestinal pseudo-obstruction. CT scan revealed **(B)** a markedly distended stomach, **(C)** dilated proximal small intestines with air–fluid levels (arrows) and edematous distal small intestines (arrowheads). Abdominal sonography revealed **(D)** duodenal dilatation, **(E)** gastric dilatation, **(F)** pelvic ascites, and a **(G)** sharp aortomesenteric angle of 14°. GB, gallbladder; DU, duodenum; SP, spleen; ST, stomach; UB, urinary bladder; UT, uterus; CX, cervix; RO, right ovary; AS, ascites.

**Figure 3:**
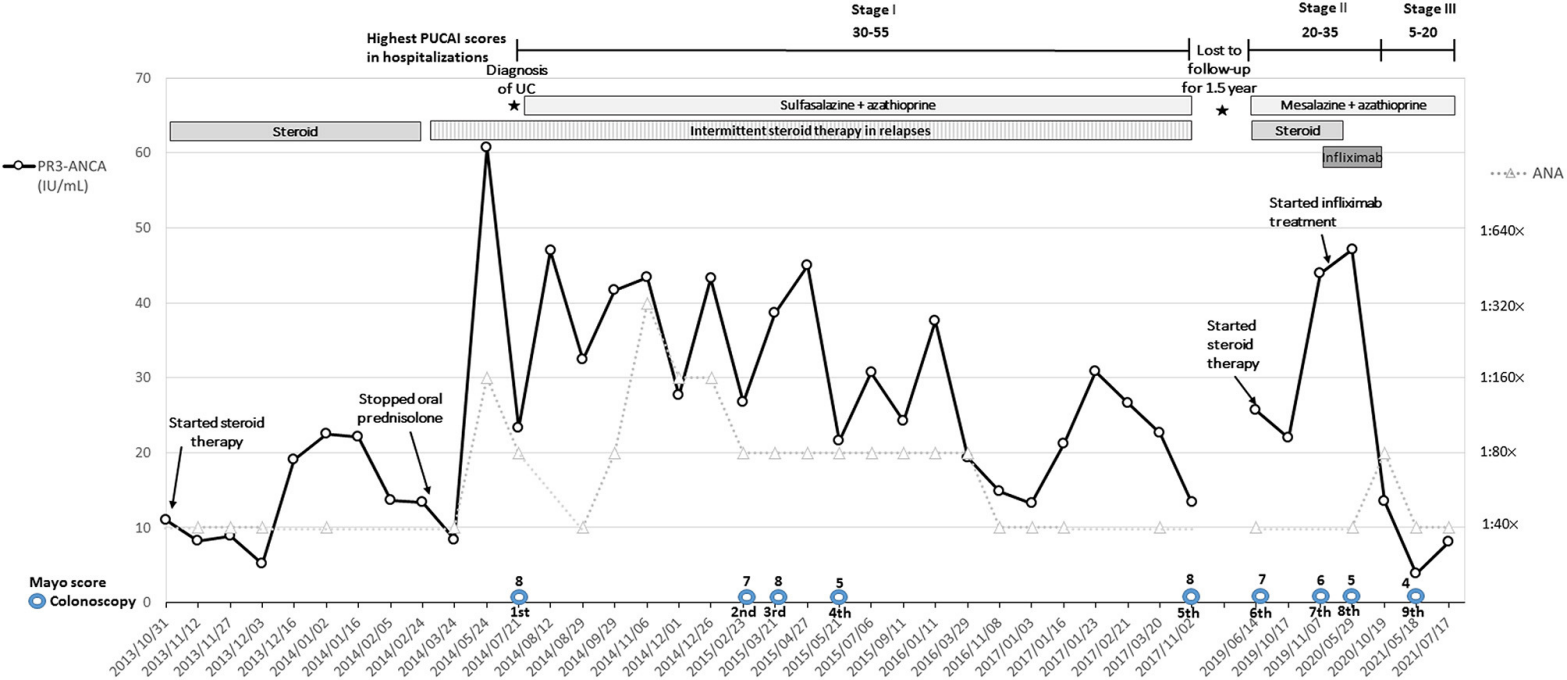
Titers of serum PR3-ANCA, ANA, ranges of highest PUCAI scores during hospitalizations, Mayo scores of nine colonoscopies, and major treatments during the three stages of the entire clinical course. PR3-ANCA titers decreased to a lower range (3.82–13.44 IU/mL, negative <2.0 IU/mL) with lessened disease activity by lowering highest PUCAI scores (5–20) during hospitalizations after infliximab treatment in combination with continuing azathioprine and mesalazine therapy.

A visibly ill 13-year-old female patient with a history of allergic urticaria to shrimp and crab and a sibling history of Moyamoya disease developed recurrent episodes of afebrile bilious vomiting, epigastralgia, and diarrhea, which all persisted for several months. The patient had been hospitalized 3 and 8 months prior to this admission and diagnosed with acute gastroenteritis twice. In addition, the patient experienced hair loss in the 6 months prior. By the third admission, the patient's weight had decreased from 38 to 34.5 kg (10th percentile); the patient had height of 153 cm (50th percentile) and a body mass index of 14.7 kg/m^2^. Physical examinations revealed sparse hair and epigastric tenderness with hypoactive bowel sounds but without rebounding pain. Abdominal radiographs showed edematous intestines with several dilated segments with air–fluid levels. Abdominal sonography revealed marked gastroduodenal dilatation with stasis and thickened intestinal mucosa. The patient was placed in *nil per os*, and ranitidine was administered. A nasogastric tube was inserted, with large volumes of greenish contents with coffee ground substances being subsequently drained. Esophagogastroduodenoscopy revealed superficial gastroduodenitis (Figures 1A,B). Pathology of the biopsy specimens revealed nonspecific foveolar hyperplasia in the gastric antrum and infiltration of the duodenal mucosa by lymphoplasma cells (Figures 1F,G). A barium follow-through examination revealed segmental dilatation at the distal jejunum (Figure 2A). Computed tomography scanning and sonography revealed gastroduodenal dilatation and mild pelvic ascites (Figures 2B–F). Laboratory data revealed neutrophilia (76.4–83.9%) in white blood cell differential counts, hypokalemia (2.9 mEq/L), positive antinuclear antibodies (ANAs 1:40 × , homogenous with cytoplasmic pattern), low C3 level (81.6 mg/dL [normal 90–180 mg/dL]), and PR3-ANCA positivity (10.99 [normal <2 IU/mL]). However, the C-reactive protein (CRP) level was <0.5 mg/dL and the fecal α1-antitrypsin concentration was <1.33 mg/g of stool [normal 0.00–2.957 mg/g]. Negative results were obtained for MPO-ANCA, anti-ds DNA antibodies, hepatic transaminases, and stool cultures. The patient was treated with intravenous methylprednisolone (1 mg/kg/day) for 6 days, followed by oral prednisolone (0.5 mg/kg/day) for approximately 3 months after achieving a stabilized condition and being discharged. Colonoscopy after 7 weeks of oral administration of prednisolone revealed pancolitis and mild rectal ulcers. Colonic pathology revealed lymphoplasma cell infiltration and inflammatory neutrophil infiltration without vasculitis. However, after oral prednisolone was discontinued, symptoms relapsed and repeated laboratory data revealed positive stool occult blood and leukocytes and fluctuating levels of decreased C3 and C4, ANA positivity (1:40–320 × , homogenous with cytoplasmic pattern), and positive PR3-ANCA with higher titers (8.41–60.69 IU/mL) than before the relapse (Figure 3). Repeated esophagogastroscopy revealed superficial gastritis and a dilated duodenum, and colonoscopy showed terminal ileitis as well as diffuse colitis with ulcers and exudates from the cecum to the rectum (Figures 1C–E). Pathology of biopsies indicated superficial capillary congestion, lymphoplasma cell infiltration of the lamina propria, loss of goblet cells, and mucin with architectural distortion from the terminal ileum to the rectum and active cryptitis with neutrophil infiltration but no vasculitis (Figures 1H–J). These findings were consistent with UC, and *in situ* PR3 immunohistochemical staining revealed PR3 positivity, mainly in the colon, focally in the duodenum, but sparsely in the terminal ileum (Figures 1K–O). The patient was then treated with sulfasalazine (1,500 mg/day), hydrocortisone enema, and mesalamine (500 mg/day) for 1 week. Symptoms fluctuated under sulfasalazine and azathioprine treatment, and the patient was hospitalized 29 times in the subsequent 3 years with highest Pediatric Ulcerative Colitis Activity Index (PUCAI) scores of 30–55 during hospitalizations (Stage I, Figure 3). The patient was lost to follow-up for 1 and half a year and was readmitted for acute weight loss (down from 48 to 45 kg) with bloody diarrhea 5 years after the diagnosis of UC relapse (highest PUCAI score during hospitalization = 35). Laboratory data revealed neutrophilia (81.6%), ANA positivity (1:40 × ), PR3-ANCA positivity (25.68 IU/mL), slightly elevated erythrocyte sedimentation rate (ESR; 21 mm/h [normal 0–20]), but normal CRP level [0.5 mg/dL (normal ≤ 0.5 mg/dL)]. Esophagastroduodenoscopy and colonoscopy revealed erosive gastritis and pancolitis, respectively. Pathology of biopsies confirmed chronic gastroduodenal inflammation without *Helicobacter pylori* infection and UC with activity. Because the patient responded poorly to 3 months of steroid therapy, 300 mg of infliximab (antitumor necrotizing factor-α antibody) was administered in eight doses over an 11-month period, during which the patient has relapsed with epigastric abdominal pain, nonbilious vomiting, diarrhea, and acute weight loss and has been hospitalized 13 times for relapsing UC with highest PUCAI scores of 20–35 during hospitalizations and fluctuating levels of ESR (17–80 mm/1 h) and CRP (0.03–1.11 mg/dL), increased fecal alpha 1-antitrypsin (7.32–7.96 mg/g of stool), weak ANA positivity (1:40–80 × ), and high PR3-ANCA levels (21.97–47.12 IU/mL) (Stage II, Figure 3). After completion of infliximab treatment, the patient's bloody diarrhea and abdominal pain were in remission with normal fecal alpha 1-antitrypsin (<1.332 mg/g of stool), weak ANA positivity (1:40 × ), and decreased PR3-ANCA levels (3.82–13.4 IU/mL) (Stage III, Figure 3). During this period, the patient maintained regular follow-ups at outpatient clinics and was orally administered sulfasalazine and azathioprine in a stable condition with good weight gain, three short hospitalizations, and no recent admission for more than 7 months.

## Discussion

A wide spectrum of diseases can present with ANCA, including ANCA-associated vasculitis (AAV) and gastroenterological pathologies (e.g., liver disease and IBD) and overlap syndromes (8). ANCAs are highly specific biomarkers of AAV, particularly in 80–90% of patients with granulomatosis with polyangiitis (formerly Wegener's granulomatosis), which has 65–70% and 20–30% rates of PR3-ANCA and MPO-ANCA positivity, respectively. The tissues commonly involved in AAV are those of the kidneys, lungs, ears, nose, throat, and, rarely, the bowels (8). ANCAs can be found in nonvasculitis conditions and chronic infections, including systemic lupus, IBD, cystic fibrosis, primary sclerosing cholangitis, and endocarditis (9). ANCAs were detected in younger patients with UC and tended to be predominant in patients with UC with left colitis or pancolitis in which the distinct subset of p-ANCA was mainly found in UC as an indicator of disease activity (10). Whether anti–*Saccharomyces cerevisiae* antibodies and ANCAs can be used as combined predictors of UC in pediatric patients with uncertified IBD remains unclear (11). Nevertheless, a study discovered that the presence of PR3-ANCA in UC was associated with more extensive colitis and shorter disease duration (12). Although the levels of PR3-ANCA in UC are lower than those in AAV (13), serum PR3-ANCA, which is more prevalent in UC than in CD, has emerged as a potential marker for accurately discriminating UC from CD (11), particularly when detection is performed using chemiluminescent immunoassays with an appropriate cut-off value (14). Furthermore, PR3-ANCA positivity can serve as a marker of disease activity and correlates with nonresponse to steroid therapy in moderate-to-severe UC and with primary nonresponse to antitumor necrotizing factor-α agents (3, 15). In our patient with nonvasculitic UC, PR3-ANCA with the highest titer of 30-fold the cut-off value was present in serum (measured through fluorescence enzyme immunoassay [FEIA], Phadia® 250, Thermo Fisher Scientific Inc., Waltham, MA, USA), and PR3 antigen was detected *in situ* in the inflamed bowels (measured through immunohistochemical staining labeled with rabbit monoclonal anti-PR3 antibody [EPR6277] (ab133613, abcam®, Cambridge, UK) to human PR3 at 1/100 dilution in formalin-fixed paraffin-embedded sections according to the user instruction). PR3 was found within the fibrinoid necrotic vasculitic glomerulus of patients with AAV (16). In our case, positive PR3 staining was discovered *in situ* in the crypt abscess of the colon, particularly in the aggregates of neutrophils, and focally in the duodenal mucosa. The serum PR3-ANCA positivity and MPO-ANCA negativity in our patient are consistent with the findings for pediatric patients with UC in other studies (6, 7). PR3-ANCA titers increased after the patient discontinued oral prednisolone and decreased after infliximab treatment, which appeared to correlate with the disease activity reported previously (3, 5). Moreover, co-occurrence of other immune-mediated inflammatory diseases—most commonly with psoriasis or asthma and rarely with other gastrointestinal diseases—was associated with poorer outcomes (17). Therefore, our patient's underlying allergic urticaria might explain the recurrent relapses and high serum PR3-ANCA titers and *in situ* PR3-ANCA staining in the bowels parallel to the pancolitis with involvement of the duodenum.

High ANA titers and poor steroid response co-occurred in the first 3-year stage after diagnosing UC of our patient, which is consistent with the findings of a previous report on the association of ANAs with steroid dependence in patients with UC (18). However, during the period of lost to follow-up for one and half a year, ANA positivity remained with low titers of 1:40 × but PR3-ANCA titers increased remarkably in correspondence to nonresponsiveness of steroid therapy after relapse (Stage II, Figure 3) and decreased to a considerably lower range after completion of infliximab treatment (Stage III, Figure 3), suggesting the superiority of PR3-ANCA to ANA titers in reflecting disease activity in our case. Because ANA positivity with high titers of ≥1:80 × was discovered during the 5th to 10th hospitalizations (reaching a titer of 1:320 × during the 8th hospitalization), systemic lupus erythematosus (SLE)/AAV overlap syndrome was initially suspected; the patient met four of the 2012 SLICC criteria for SLE: nonscarring alopecia, serositis, ANAs, and low complement C3. Low levels of circulating C3, found in 5–20% of patients with active AAV, are associated with poorer outcomes (9). However, the role of decreased C3 levels in non-AAV UC remains unclear. SLE/AAV overlap syndrome has mostly been found in women and usually presents with MPO-ANCA positivity. However, the role of PR3-ANCA in SLE is less established, and reports regarding the association between UC and SLE are rare (11, 19). Moreover, our case demonstrated neutrophilia (66–96%) without remarkable elevation of CRP level (0.5–2.1 mg/dL) despite having increased PUCAI scores of 35–40 during most hospitalizations, which suggests that neutrophilia may be an early sign of PR3-ANCA–associated UC. In one study, high CRP and MPO-ANCA positivity were found in a teenager with comorbid UC and AAV with involvement of the skin, bowel, and peripheral nerves (20). By contrast, our case demonstrated PR3-ANCA positivity in serum and *in situ*, ANA positivity, and low CRP levels with the absence of AAV; overlapping UC and SLE (or other autoimmune diseases) could not be excluded from consideration. Fecal calprotectin is useful for evaluating disease activity in UC (21), particularly as a marker of gut inflammation when associated with any extraintestinal manifestations such as enthesitits related arthritis (22). However, neither arthralgia nor joint stiffness developed in our patient and fecal calprotectin was not checked because of its unavailability. Instead, fecal alpha 1-antitrypsin was used as an alternative to evaluate the patient's intensity of protein-losing enteropathy.

Pancolitis with involvement of the UGI tract is more common in children with UC than in adults with UC (23). In our case, recurrent bilious vomiting was the initial major clinical manifestation of UC and may have been due to multiple etiologies of intestinal pseudo-obstruction. First, the patient's backwash ileitis (BWI) was verified through colonoscopy and proximal segmental dilatation of the jejunum in the barium follow-through examination. Nonspecific mucosal inflammation in the terminal ileum (e.g., BWI) was found in 10–20% of patients with UC; furthermore, pathogenesis of BWI may be associated with reflux of colonic contents into the terminal ileum (21), which may present as ileocecal valve gaping and terminal ileum dilatation in magnetic resonance enterography (24). Second, follow-up abdominal sonography revealed an aortomesenteric angle of 14° (Figure 2G), indicating partial obstruction of the third portion of the duodenum and meeting the criteria of an angle <22° for superior mesenteric artery (SMA) syndrome (25). Our patient's low body weight with a low body mass index and SMA syndrome worsened by repeated episodes of acute weight loss further exacerbated the bilious vomiting. Third, the recurrent bilious vomiting could have been induced by paralytic ileus from the hypokalemia or the gastroduodenitis revealed through esophagogastroduodenoscopy, computed tomography, and sonography. Fourth, the recurrent bilious vomiting could have multifactorial etiologies, including PR3-ANCA-associated gastroenteritis, stress-induced gastroduodenitis, and other gastroenteropathy-causing ascites. However, further clinical cases are required to elucidate whether PR3-ANCA plays a role in gastroduodenitis as a subset of UC.

To our knowledge, this is the first report of a pediatric patient with *in situ* pathological evidence of PR3 without vasculitis in the bowels after diagnosis of PR3-ANCA–associated UC and with rare initial presentation of intestinal pseudo-obstruction-induced recurrent bilious vomiting. Notably, the patient's highly elevated serum PR3-ANCA titers (>10 to 30-fold of upper limit of normal) appeared in coincidence with the increased disease activity and nonresponse to steroid therapy; such high PR3-ANCA titers decreased to a much lower range (<10-fold of upper limit of normal) with lessened disease activity after infliximab treatment and the PR3-ANCA titers remained within such a low range compatible with her stable condition for more than 2 years (Figure 3). What role PR3 plays in the pathogenesis of UC and whether the clinical features of the present case constitute overlap syndrome with other autoimmune disease or a disease variation of UC warrant further investigation.

## Data Availability Statement

The original contributions presented in the study are included in the article/supplementary material, further inquiries can be directed to the corresponding author/s.

## Ethics Statement

The studies involving human participants were reviewed and approved by the Taipei Medical University-Joint Institutional Review Board (TMU-JIRB) (No. N201602030). Written informed consent to participate in this study was provided by the participants' legal guardian/next of kin. Written informed consent was obtained from the minor(s)' legal guardian/next of kin for the publication of any potentially identifiable images or data included in this article.

## Author Contributions

C-WY summarized the medical records, wrote the initial manuscript, and approved the final manuscript as submitted. Y-CK performed the pathological examination and staining of the biopsied gastrointestinal tissues, and revised the manuscript. P-CL edited the medical and pathological images, and revised the final manuscript as submitted. H-YC performed the esophagogastroduodenoscopy and colonoscopy. S-CL provided his expertise in profiles of autoimmune diseases and consultation advice during hospitalization. Y-HL was the first-line pediatrician during the patient's hospitalization and summarized the medical records of the patient. Y-LH performed the barium follow-through examination and CT scan with their interpretation. S-BF organized the team to treat and diagnose the patient, and revised the final manuscript as submitted. All authors contributed to manuscript revision, read and approved the submitted version.

## Conflict of Interest

The authors declare that the research was conducted in the absence of any commercial or financial relationships that could be construed as a potential conflict of interest.

## Publisher's Note

All claims expressed in this article are solely those of the authors and do not necessarily represent those of their affiliated organizations, or those of the publisher, the editors and the reviewers. Any product that may be evaluated in this article, or claim that may be made by its manufacturer, is not guaranteed or endorsed by the publisher.
